# Impact of Sacral Surface Therapeutic Electrical Stimulation on Early Recovery of Urinary Continence after Radical Retropubic Prostatectomy: A Pilot Study

**DOI:** 10.1155/2010/102751

**Published:** 2010-04-29

**Authors:** Haruo Nakagawa, Yasuhiro Kaiho, Shunichi Namiki, Shigeto Ishidoya, Seiichi Saito, Yoichi Arai

**Affiliations:** Department of Urology, Graduate School of Medicine, Tohoku University, 1.1 Seiryo Aoba-ku Sendai 980-8574, Japan

## Abstract

*Objectives*. To investigate whether sacral surface therapeutic electrical stimulation (SSTES) initiated during the early postoperative period would be effective towards early recovery of postprostatectomy urinary continence. *Methods*. A total of 35 consecutive patients who underwent radical prostatectomy by a single surgeon were enrolled in this study. Twenty early patients began pelvic floor muscle exercise (PME). Fifteen subsequent patients received SSTES postoperatively with no instruction for PME provided. Immediate urinary function just after catheter removal was evaluated with frequency-volume chart and 24-hour pad test. *Results*. There were no differences between the SSTES and PME groups in maximum voided volume capacity (MVV) and urine loss ratio (ULR) on the first day after removal of urethral catheter. However, on day 3 MVV was significantly larger and ULR was also significantly lower in the SSTES group. *Conclusions*. SSTES treatment is feasible and appears to be effective for early recovery of urinary continence after radical prostatectomy.

## 1. Introduction

Urinary incontinence and sexual dysfunction are representative long-term complications of radical prostatectomy. During several months after radical prostatectomy, urinary incontinence develops in most patients, significantly lowering their quality of life (QOL). One year after the surgery, the incidence of urinary incontinence, the ratio of patients who require pads, and the ratio of those who experience urinary incontinence, even if only slightly, reach 5%–15%, 33%, and 66%, respectively [1, 2]. The main mechanism of postprostatectomy incontinence is considered to be damage of the sphincter muscle caused upon separation of the prostate and urethra. It has been clarified in recent years that preservation of the neurovascular bundle could be involved in the recovery of urinary continence [3], and thus a neurogenic mechanism for the sphincter muscle was indicated. In addition, detrusor overactivity can develop as a consequence of traction of the bladder. It has been reported that urinary incontinence related to detrusor overactivity occurs in 40% and detrusor overactivity incontinence occurs in 13% of patients after radical prostatectomy [4]. To improve the postoperative urinary continence, we should consider not only urinary sphincter muscle damage but also bladder-related factors.

Pelvic floor exercise (PME) has been widely performed after radical prostatectomy with the aim of the prevention and treatment of urinary incontinence. The effectiveness of the PME depends on its instruction method, and recent reports have suggested that the effectiveness would become higher by using the preoperative biofeedback method [5].

Neuromodulation, using electrical or magnetic stimulation, was developed for urgency incontinence as well as stress incontinence [6]. These methods were used to treat postprostatectomy incontinence (PPI) [7–10]. A certain effect was observed in these studies, while these studies were applied only for patients with urinary incontinence. We developed sacral surface therapeutic electrical stimulation (SSTES) as a therapy for urinary incontinence using neuromodulation [11]. In this therapy, skin surface electrodes are applied on the sacral surface to provide stimulation, making the treatment very easy to perform. It has been shown that SSTES has not only an inhibitory effect on detrusor overactivity but also an efferent stimulant effect to the pudendal nerve [11]. It is thus expected that SSTES initiated in an early postoperative period would be effective for early recovery of postoperative urinary continence.

## 2. Materials and Methods

**Figure 1:**
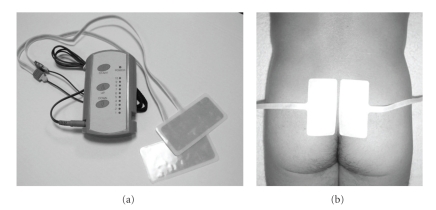
Portable electrical stimulator (Nodoka, Lintec Co. Ltd., Tokyo, Japan) and a pair of the specially-designed electrodes. A pair of electrodes was placed symmetrically on the skin surface over the second through fourth posterior sacral foramens.

The study population consisted of 40 consecutive patients who underwent retropubic radical prostatectomy for newlydiagnosed, clinicallylocalized prostate cancer from November 2004 to November 2006. All of the operations were performed by a single surgeon (Y.A.) and technical modifications were not made except smaller skin incision during the study period. This surgeon experienced more than 500 radical retropubic prostatectomy procedures over 20 years. The patients with prolonged indwelling urethral catheter due to anastomotic leakage (two cases) and who required reinsertion of the urethral catheter because of transient dysuria (one case) were excluded from the study, since the long duration of the indwelling urethral catheter might affect the immediate continence postcatheter removal. The patients who could not complete the frequency volume chart (one case) and withdrew his consent for the use of the SSTES (one case) were also excluded. Thus, a total of 35 patients were enrolled in the study. Among them, 20 early patients (from November 2004 to December 2005) received instruction for the PME and began the exercise one day before the surgery and continued for 1 week or longer. Fifteen subsequent patients (from January 2006 to November 2006) received SSTES, which was started at postoperative day 1 with no PME instruction provided. None of the patients were prescribed anticholinergic drugs during this study. For SSTES, the stimulator was specially designed for this purpose (Nodoka, Lintec Co. Ltd., Tokyo, Japan) (Figure 1), and a pair of speciallydesigned plate electrodes with a contact surface 4-cm × 9-cm (width × height; Electrode type A, Lintec, Tokyo, Japan) (Figure 1) were placed symmetrically on the skin surface over the second through fourth posterior sacral foramens. Pulses of 30-Hz frequency at 200-*μ*s pulse width and maximum output of 80 V were used for 15 minutes twice a day for 1 week. Intensity was controlled by each patient below the pain threshold. The urethral catheter was removed on day 5 or 6 postsurgery. Immediate urinary function just after catheter removal was evaluated with a daily frequency-volume chart and 24-hour pad test. The urine loss ratio [12] was defined as the weight of urine loss in the pad divided by the daily urine volume, that is, micturition volume plus incontinence volume. The maximum voided volume (MVV) was defined as largest voided volume during single micturition from daily frequency-volume chart. The Ethics Committee of the Tohoku University School of Medicine approved this study and informed consent was obtained from all of the patients. Statistical software (JMP Statistical Discovery Software, SAS Institute, Cary, NC, USA) was used for all analyses. Tested groups were compared by unpaired Student's *t*-test; *P*-values <.05 were considered statistically significant.

## 3. Results

Table 1 presents the patient demographics and pathological characteristics. There were no significant differences in any of the baseline clinical or pathologic parameters between the two groups: age, PSA, tumor stage, biopsy Gleason score, or degree of nerve preservation. No differences were observed in any of the postoperative parameters: operative time, estimated blood loss, prostate weight, pathological stage, or positive surgical margin status. During hospitalization, each group received the scheduled PME or SSTES treatment, respectively, under the instruction of the nursing staff. One patient in the SSTES group, who experienced sinus tachycardia and discomfort during electrical stimulation, with a fever of 38 degrees centigrade and expressed a desire to stop electrical stimulation, was excluded from the analysis.

**Figure 2:**
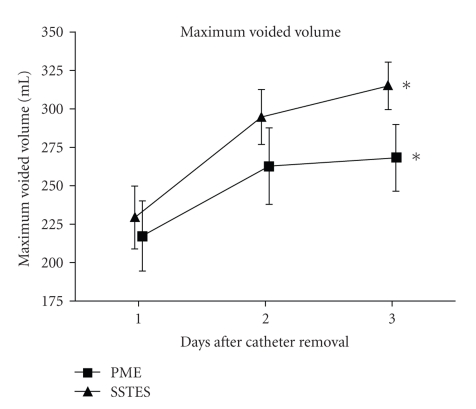
Maximum voided volume at day 1, 2, and 3 after removal of the urethral catheter. Error bars represent SEs. *P < .05.

**Figure 3:**
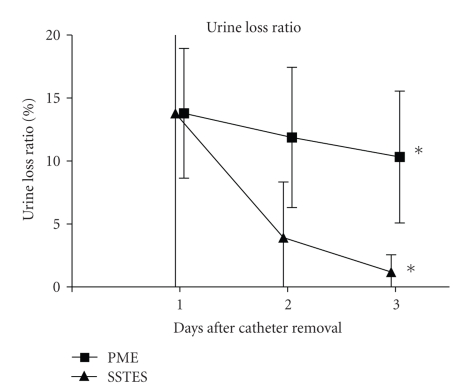
Percentage of urine loss ratio at day 1, 2, and 3 after removal of the urethral catheter. The urine loss ratio was defined as the weight of urine loss in the pad divided by the daily urine volume. Error bars represent SEs. *P < .05.

On the first day after removal of the urethral catheter, there were no significant differences between the SSTES and PME groups in maximum voided volume (229.3 ± 79.2 ml (mean±standard deviation) versus 217.4 ± 99.5 ml, resp.; *P* = .35) or urine loss ratio (13.8 ± 19.9% versus 14.5 ± 23.7%, resp.; *P* = .46). However, the maximum voided volume and urine loss ratio were rapidly improved in the SSTES group (Figures 2 and 3). On the third day after removal of the urethral catheter, the maximum bladder capacity was significantly larger in the SSTES group than in the PME group (315.0 ± 59.9 ml versus 268.1 ± 94.6 ml, resp.; *P* < .05). Urine loss ratio was also significantly lower in the SSTES group (1.18 ± 1.36% versus 10.32 ± 22.7%, *P* < .05). During the study period, there were no significant adverse effects observed, except for the one case described above. No patients showed the symptom of difficulty on urination. No patients complained dysuria or urinary retention during the hospital stay.

## 4. Discussion

In the literature, urinary incontinence is one cause of lowering the quality of life for patients following radical prostatectomy. Many efforts, including neuromodulation, have been made to achieve early urinary continence. Yokoyama et al. reported that extracorporeal magnetic stimulation improved continence in 60% of patients with PPI [9, 10]. On the other hand, two randomized control studies failed to show an additional effect of neuromodulation for PPI compared with PME alone [7, 8]. In these studies, electrical stimulation was applied with an anal surface electrode and was initiated after urethral catheter removal. Furthermore, in all of the studies noted above, neuromodulatory stimulation was applied in patients with existing urinary incontinence. It is well known that early rehabilitation has an advantage for early and satisfactory functional recovery in nonurological fields, such as orthopedics [13], cardiac surgery [14], neurosurgery [15], and spinal cord trauma [16]. PPI occurs due to surgical damage of the urethral sphincter, pelvic floor muscle, and bladder. From this perspective, we generated the idea of initiating electrical stimulation on the first day after radical prostatectomy. As a result of this early electrical rehabilitation, significant effects on early recovery of continence and maximum voided volume were observed. To our knowledge, this is the first report on the possible rehabilitative role of neuromoduration for PPI. 

The present study showed the possible utility of SSTES for early recovery of urinary function following radical prostatectomy. We previously shown that postcatheter removal incontinence is significantly related to postoperative urinary function after radical prostatectomy [17]. Therefore, it is expected that minimizing the postcatheter removal incontinence could ultimately affect the postoperative urinary quality of life. In this study, on the first day after catheter removal, there were no differences in maximum voided volume or urine loss ratio between the SSTES and PME groups. On the other hand, on the third day after catheter removal, the maximum voided volume and urine loss ratio rapidly improved with SSTES. 

It has been reported that SSTES exhibits not only an inhibitory effect on detrusor overactivity but also an efferent stimulant effect via the pudendal nerve [11]. The effect may be partly due to decreasing urgency with electrical stimulation [18]. Pelliccioni and scarpino reported the external anal sphincter response with S3 surface electrical stimulation [19]. Indeed, we macroscopically observed that contraction of the pelvic floor muscle including the urethral sphincter and levator muscle was synchronized with SSTES during open radical prostatectomy (data not shown). The efferent effect on the pudendal nerve, which is equivalent to the effect of pelvic floor muscle exercise, and the afferent inhibitory effect can be expected for injured pelvic floor muscle and detrusor overactivity that develops after radical prostatectomy. Based on the results of this study, SSTES appears to have an early rehabilitative role on postprostatectomy urinary function. 

We acknowledge several limitations in this pilot study. First, our study had relatively few patients. Second, although there was no statistical difference, the prostate volume and blood loss were smaller in the SSTES group, which may have influenced the results. Third, the study was not performed in a randomized fashion but as a historical control study. However, all operations were performed by a single, well-experienced surgeon and technical modifications were not made during the study that might minimize the intraoperator's bias, such as a learning effect. Fourth, one of the drawbacks of the neuromodulatory approach is the short carry-over effect. It is unknown whether 1-week of electrical stimulation could affect the recovery of urinary function for 1 month or longer after surgery. Indeed, it was difficult to accurately evaluate urine loss ratio using the 24-hour pad test on an outpatient basis. In the present pilot study, the optimal duration of SSTES remains to be elucidated. Nevertheless, the results show the possible rehabilitative role of SSTES in the early phase of recovery of urinary function following radical prostatectomy 

A multi-institutional, randomized controlled study with a large number of subjects is now on going.

## 5. Conclusion

 We investigated the rehabilitative role of SSTES for recovery of urinary function following radical prostatectomy. This treatment is feasible and appears to be effective for early recovery of urinary continence after surgery. A randomized controlled trial with a large study population is warranted to confirm its effectiveness.

## Figures and Tables

**Table 1:** The demographic and clinical characteristics of the patient population.

	PFE (*n* = 20)	SS- TES (*n* = 15)	P*
Age (year)	63.1 ± 6.4	61.4 ± 6.6	.46
PSA	10.7 ± 9.1	9.8 ± 6.6	.72
Clinical stage			

T1	70%	80%	
T2	15%	13%	
T3	15%	7%	.72 c
Biopsy Gleason sum			

6	5%	7%	
7	85%	86%	
8	5%	7%	
9	5%	0%	.84 c
Nerve sparing			

No nerve sparing	10%	13%	
Unilateral	50%	33%	
Bilateral	40%	53%	.62 c

Operation time	221 ± 66	226 ± 71	.85
Blood loss	1170 ± 721	838 ± 360	.11
Prostate weight	59.7 ± 30.1	45.1 ± 12.8	.09

Pathological stage			
pT2	80%	77%	
pT3	20%	23%	.74 c
Positive margin	20%	13%	.60 c

c: Chi-square test.

*Unpaired *t*-test unless otherwise noted.

## References

[1] Namiki S, Tochigi T, Kuwahara M (2004). Recovery of health related quality of life after radical prostatectomy in Japanese men: a longitudinal study. *International Journal of Urology*.

[2] Namiki S, Terai A, Nakagawa H (2005). Intraoperative electrophysiological confirmation of neurovascular bundle preservation during radical prostatectomy: long-term assessment of urinary and sexual function. *Japanese Journal of Clinical Oncology*.

[3] Kaiho Y, Nakagawa H, Ikeda Y (2005). Intraoperative electrophysiological confirmation of urinary continence after radical prostatectomy. *Journal of Urology*.

[4] Huckabay C, Twiss C, Berger A, Nitti VW (2005). A urodynamics protocol to optimally assess men with post-prostatectomy incontinence. *Neurourology and Urodynamics*.

[5] Burgio KL, Goode PS, Urban DA (2006). Preoperative biofeedback assisted behavioral training to decrease post-prostatectomy incontinence: a randomized, controlled trial. *Journal of Urology*.

[6] Yamanishi T, Kamai T, Yoshida K-I (2008). Neuromodulation for the treatment of urinary incontinence. *International Journal of Urology*.

[7] Moore KN, Griffiths D, Hughton A (1999). Urinary incontinence after radical prostatectomy: a randomized controlled trial comparing pelvic muscle exercises with or without electrical stimulation. *BJU International*.

[8] Wille S, Sobottka A, Heidenreich A, Hofmann R (2003). Pelvic floor exercises, electrical stimulation and biofeedback after radical prostatectomy: results of a prospective randomized trial. *Journal of Urology*.

[9] Yokoyama T, Nishiguchi J, Watanabe T (2004). Comparative study of effects of extracorporeal magnetic innervation versus electrical stimulation for urinary incontinence after radical prostatectomy. *Urology*.

[10] Yokoyama T, Inoue M, Fujita O, Nozaki K, Nose H, Kumon H (2005). Preliminary results of the effect of extracorporeal magnetic stimulation on urinary incontinence after radical prostatectomy: a pilot study. *Urologia Internationalis*.

[11] Yokozuka M, Namima T, Nakagawa H, Ichie M, Handa Y (2004). Effects and indications of sacral surface therapeutic electrical stimulation in refractory urinary incontinence. *Clinical Rehabilitation*.

[12] Ates M, Teber D, Gozen AS (2007). A new postoperative predictor of time to urinary continence after laparoscopic radical prostatectomy: the urine loss ratio. *European Urology*.

[13] Kjellby-Wendt G, Styf J, Carlsson SG (2001). Early active rehabilitation after surgery for lumbar disc herniation: a prospective, randomized study of psychometric assessment in 50 patients. *Acta Orthopaedica Scandinavica*.

[14] Macchi C, Fattirolli F, Lova RM (2007). Early and late rehabilitation and physical training in elderly patients after cardiac surgery. *American Journal of Physical Medicine and Rehabilitation*.

[15] Barbara M, Monini S, Buffoni A (2003). Early rehabilitation of facial nerve deficit after acoustic neuroma surgery. *Acta Oto-Laryngologica*.

[16] Tederko P, Limanowska H, Krasuski M, Kiwerski J (2006). Problems of adaptation to wheelchair in early stage rehabilitation after spinal cord trauma. *Ortopedia Traumatologia Rehabilitacja*.

[17] Saito S, Namiki S, Numahata K (2006). Relevance of postcatheter removal incontinence to postoperative urinary function after radical prostatectomy. *International Journal of Urology*.

[18] Walsh IK, Johnstone RS, Keane PF (1999). Transcutaneous sacral neurostimulation for irritative voiding dysfunction. *European Urology*.

[19] Pelliccioni G, Scarpino O (2006). External anal sphincter responses after S3 spinal root surface electrical stimulation. *Neurourology and Urodynamics*.

